# Surgeon volume and body mass index influence positive surgical margin risk after robot-assisted radical prostatectomy: Results in 732 cases

**DOI:** 10.1080/2090598X.2019.1619276

**Published:** 2019-05-30

**Authors:** Antonio B. Porcaro, Alessandro Tafuri, Marco Sebben, Paolo Corsi, Tania Processali, Marco Pirozzi, Nelia Amigoni, Riccardo Rizzetto, Aliasger Shakir, Giovanni Cacciamani, Arianna Mariotto, Matteo Brunelli, Riccardo Bernasconi, Giovanni Novella, Vincenzo De Marco, Walter Artibani

**Affiliations:** a Department of Urology, University of Verona, Azienda Ospedaliera Universitaria Integrata Verona, Verona, Italy; b Catherine & Joseph Aresty Department of Urology, USC Institute of Urology, Keck School of Medicine, University of Southern California (USC), Los Angeles, CA, USA; c Department of Pathology, University of Verona, Azienda Ospedaliera Universitaria Integrata Verona, Verona, Italy

**Keywords:** Prostate cancer, radical prostatectomy, robotic surgery, positive surgical margins, body mass index

## Abstract

**Objectives**: To evaluate clinicopathological and perioperative factors associated with the risk of focal and non-focal positive surgical margins (PSMs) after robot-assisted radical prostatectomy (RARP).

**Patients and methods**: The study was retrospective and excluded patients who were under androgen-deprivation therapy or had prior treatments. The population included: negative SM cases (control group), focal and non-focal PSM cases (study groups). PSMs were classified as focal when the linear extent of cancer invasion was ≤1 mm and non-focal when >1 mm. The independent association of factors with the risk of focal and non-focal PSMs was assessed by multinomial logistic regression.

**Results**: In all, 732 patients underwent RARP, from January 2013 to December 2017. An extended pelvic lymph node dissection was performed in 342 cases (46.7%). In all, 192 cases (26.3%) had PSMs, which were focal in 133 (18.2%) and non-focal in 59 (8.1%). Independent factors associated with the risk of focal PSMs were body mass index (odds ratio [OR] 0.914; *P *= 0.006), percentage of biopsy positive cores (BPC; OR 1.011; *P* = 0.015), pathological extracapsular extension (pathological tumour stage [pT]3a; OR 2.064; *P* = 0.016), and seminal vesicle invasion (pT3b; OR 2.150; *P* = 0.010). High surgeon volume was a protective factor in having focal PSM (OR 0.574; *P* = 0.006). Independent predictors of non-focal PSMs were BPC (OR 1,013; *P* = 0,044), pT3a (OR 4,832; *P* < 0.001), and pT3b (OR 5,153; *P* = 0.001).

**Conclusions**: In high-volume centres features related to host, tumour and surgeon volume are factors that predict the risk of focal and non-focal PSMs after RARP.

**Abbreviations:** AJCC: American joint committee on cancer; AS: active surveillance; ASA: American society of anesthesiologists; BCR: biochemical recurrence; BMI: body mass index; BPC: percentage of biopsy positive cores; ePLND: extended lymph node dissection; H&E: haematoxylin and eosin; IQR, interquartile range; ISUP: international society of urologic pathology; LNI: lymph node invasion; LOS: length of hospital stay; mpMRI: multiparametric MRI; (c)(p)N: (clinical) (pathological) nodal stage; OR: odds ratio; OT: operating time; PSA-DT: PSA-doubling time; (P)SM: (positive) surgical margin; (NS)(RA)RP: (nerve-sparing) (robot-assisted) radical prostatectomy; RT: radiation therapy; (c)(p)T: (clinical) (pathological) tumour stage

## Introduction

Prostate cancer is the most common non-cutaneous malignancy and the second leading cause of cancer-related deaths in men [1]. The disease requires a clinical risk evaluation [2] in order to plan suitable management options, which include: active surveillance (AS), radical prostatectomy (RP), and radiation therapy (RT) [3]. Today, RP is performed more frequently using the robot-assisted RP (RARP) approach. An unfavourable outcome after RP is the detection of positive surgical margins (PSMs), which expose patients to the risk of a second treatment including adjuvant or salvage RT, with or without androgen blockade because of disease recurrence [3–10]. High-grade tumours extending beyond the prostate with a PSM and PSA-doubling time (PSA-DT) of <3 months represent negative prognostic factors for metastases and prostate cancer-specific mortality [4,11,12]. Patients who have extraprostatic disease with PSMs need appropriate counselling for further management options that include: immediate RT (after recovery of the urinary function) or close PSA monitoring with salvage RT before the PSA level approaches values >0.1 ng/mL [3]. These issues impair the quality of life of the affected patients, due not only to anxiety but also the toxicities related to treatments [3].

The unfavourable outcome of detecting PSMs after RP depends on both tumour biology and surgeon experience [3–10]. In high-volume centres, experienced surgeons can achieve comparable PSM rates [13]. In contemporary patient cohorts undergoing RARP, it is important to assess factors associated with the risk of PSM by the linear extent, which has prognostic value for disease recurrence [3–10]. The aim of the present study was to evaluate clinicopathological and perioperative factors associated with the risk of focal and non-focal PSMs in a contemporary cohort of patients undergoing RARP in a high-volume centre.

## Patients and methods

### Study features

The study was retrospective, had Institutional Review Board approval, and included the period from January 2013 to December 2017. Each patient provided informed-signed consent for use of data. All patients who were under androgen blockade and/or had prior treatments were excluded.

### Clinical features

Preoperative patient data included age and body mass index (BMI; kg/m^2^). The serum levels of PSA (ng/mL) were determined by radioimmunoassay. Prostate biopsies had the following features: (i) at least 12–14 cores; (ii) number of positive cores; (iii) measurement of prostate volume (mL); (iv) tumour classified by biopsy grade groups according to the 2014 International Society of Urologic Pathology (ISUP) system [14,15]. In each patient, the number of positive cores over the total number of cores taken (percentage of biopsy positive cores [BPC]) was evaluated. Patients were clinically staged for the tumour (cT) and nodal (cN) status using the 2010 American Joint Committee on Cancer (AJCC) staging system for prostate cancer (7th edition) [16]. Tumours were staged by DRE and/or by multiparametric MRI (mpMRI). Pelvic lymph nodes were assessed by CT or mpMRI. Enlarged pelvic nodes measuring >1 cm in diameter along its longest axis were staged as cN1. The metastatic status was assessed by CT and/or mpMRI, as well as by skeletal scintigraphy. Patients were then classified according to D’Amico risk classes [2].

Extended lymph node dissection (ePLND) was performed when the risk of lymph node invasion (LNI) was >5% [17]. In low-risk patients, the decision to perform an ePLND was based on clinical factors indicating increased risk of tumour upgrading in the surgical specimen [18–23].

### Perioperative features

RARP was performed using the da Vinci® Robot System (Intuitive Surgical Inc., Sunnyvale, CA, USA) via a transperitoneal approach, with antegrade prostatic dissection [24]. Nerve-sparing RP (NSRP) was undertaken when indicated [25]. According to the nerve-sparing status, the prostate was dissected by an intrafascial, interfascial or extrafascial technique [26]. RARP was performed by five experienced surgeons using a bladder neck-sparing technique [27]. All of these surgeons completed the RARP learning curve before the beginning of the present patients’ enrolment. The single high-volume surgeon had already performed >500 RARPs when patient enrolment started. The other four surgeons (low-volume surgeons) had already performed between 50 and 60 procedures at the commencement of patient enrolment.

The high-volume surgeon (W.A.) performed two-thirds of the procedures. Intraoperatively, operating time (OT, min) and blood loss (mL) were measured. Preoperatively, patients’ surgical risk was evaluated using the American Anesthesiologists Score (ASA) system [28]. Postoperatively, the length of hospital stay (LOS) was recorded in each patient who was assessed and followed for a period of 6 months, to detect hospital readmission and complications, which were classified according to the Clavien–Dindo system [29].

### Pathological features

Specimens were processed according to the Stanford protocol by dedicated pathologists [20]. Prostate weight (g) was calculated and tumours were classified according to the ISUP pathological grade group system [14,15]. Nodal packets were grouped according to a standard template and submitted in separate packages. Lymph nodes were histopathologically assessed after haematoxylin and eosin (H&E) staining. Immunohistochemical staining was performed if appropriate. In every case, the numbers of removed and metastatic nodes were evaluated. Specimens were staged using the 2010 AJCC staging system for prostate cancer (pathological tumour stage [pT] and pathology nodal stage [pN] status).

**Figure 1.**
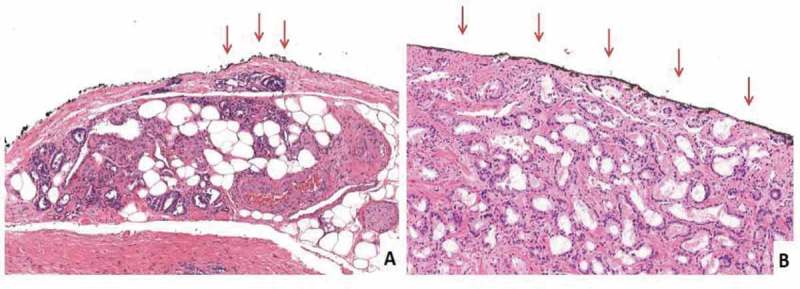
Histopathological images of focal and non-focal PSMs. The arrows indicate the PSM site. (A) The inked margin has neoplastic cells with a length ≤1 mm, i.e. focal PSM (H&E, ×10). (B) The inked margin has neoplastic cells with a length >1 mm, i.e. non-focal (in the present case, the entire inked margin is involved. H&E, ×10).

SMs were considered positive when cancer invaded the inked surface of the specimen. When the linear extension of cancer involvement on the inked surface was ≤1 mm the PSM was classified as focal, otherwise it was coded as non-focal (Figure 1). According to the anatomical location, PSMs were classified as apical, posterolateral (left and right), posterior, anterior, and bladder neck.

**Figure 2.**
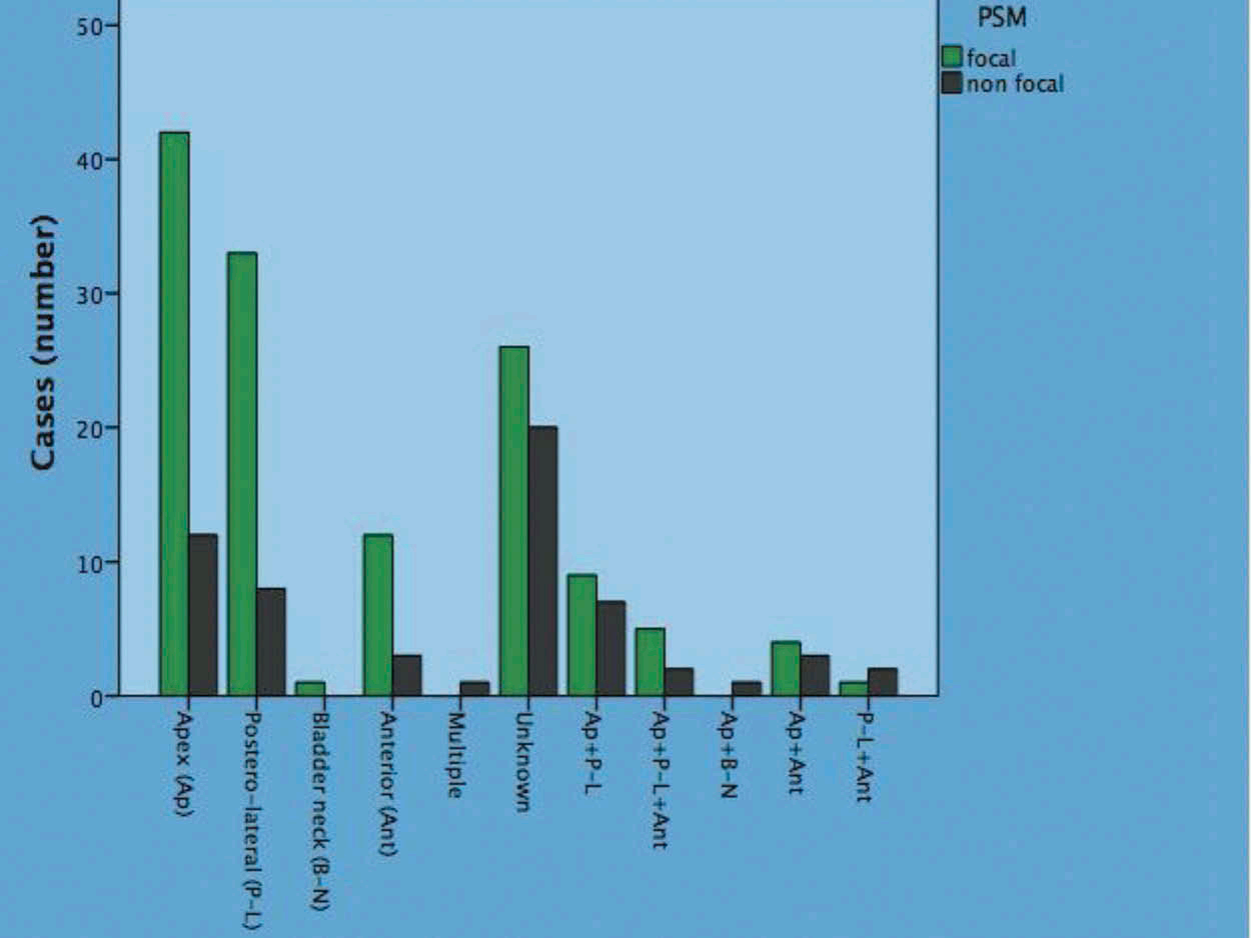
Anatomical sites of PSMs by linear extent (focal/non-focal) in 192 cases.

### Study design

The patient population was divided into three groups including: negative SM cases (no PSM; control group), as well as focal and non-focal PSM patients (study groups). The associations of focal and non-focal PSMs with clinicopathological and perioperative factors were evaluated. Clinical variables assessed included: age, BMI, PSA level, prostate volume, BPC, biopsy grade group, cT stage, cN stage, and D’Amico’s class risk. The pathological factors assessed included: prostate weight, pathological grade group, pT, and pN. Perioperative factors assessed included: OT, blood loss, ePLND, number of dissected nodes, NSRP, surgeon volume, ASA score, LOS, Clavien–Dindo score, and readmission.

### Statistical analysis

Summary statistics and distributions of factors amongst the three groups (negative SM, focal PSM, and non-focal PSM) were assessed. Data on continuous variables were reported as medians with their respective interquartile ranges (IQRs). Data on categorical variables were presented as frequencies with relative percentages. Associations of factors amongst the three groups were analysed by the Kruskal–Wallis test for continuous variables, and by the Pearson’s chi-squared test or Fisher’s exact test, as appropriate for categorical ones. Significant factors were entered into the multivariate model. The multinomial logistic regression model (multivariate analysis) evaluated the associations between factors and the risk of focal and non-focal PSMs when compared to negative SM cases (control group). Clinical, pathological and perioperative factors were evaluated separately. An overall multivariate model including independent factors of each set (clinical, pathological and perioperative) was also computed.

The software used for the analysis was the IBM Statistical Package for the Social Sciences (SPSS®), version 20 (SPSS Inc., IBM Corp., Armonk, NY, USA). All tests were two-sided with *P *< 0.05 considered to indicate statistical significance.

## Results

We evaluated 732 patients who underwent RARP (Table 1). The median age of the patients was 65 years and the median PSA level was 6.3 ng/mL. The intermediate-risk class included 50.1% of the patients. The remaining population encompassed low-risk (34.2%) and high-risk patients (15.7%). Extraprostatic extension was present in 21.9% of patients and showed high-grade cancer (pathological grade group 4–5) in 19.5% of patients. Amongst the 342 patients who had an ePLND, LNI was detected in 49 (14.3%). The median number of dissected nodes was 26. In patients with LNI, the median (IQR) number of positive nodes was 1 (1–3). The high-volume surgeon performed 66.1% of the procedures. NSRP was performed in 82% of the patients. Major complications (Clavien–Dindo score >2) were detected in 2.9% of the patients.

**Table 1.** Associations of factors with PSM in the 732 patients who underwent RARP.

		SMs			
Variable	Population	Negative	Focal PSM	Non-focal PSM	*P*
Number of patients, (%)	732	540 (73.8)	133 (18.2)	59 (8.1)	
**Clinical factors**					
Age, years, median (IQR)	65 (60–69)	65 (60–69)	65 (61–70)	62 (57–68)	0.089
BMI, kg/m^2^, median (IQR)	25.8 (23.8–28)	26 (24–28)	25.1 (23.3–27.2)	25.3 (23.4–28.4)	0.035
PSA, ng/mL, median (IQR)	6.3 (4.9–8.7)	6.1 (4.8–8.3)	6.9 (5.1–8.9)	6.9 (5.1–11.3)	0.007
Prostate volume, mL, median (IQR)	39 (30–50)	40 (30–50)	39 (30–48.8)	35 (27–45)	0.202
BPC, %, median (IQR)	29 (17–45.7)	28 (17–42)	33 (21–50)	35 (21–57)	<0.001
Clinical T stage, *n* (%)					
cT1c	517 (70.6)	390 (72.2)	83 (62.4)	44 (74.6)	0.232
cT2	194 (26.5)	135 (25)	45 (33.8)	14 (23.7)	
cT3	21 (2.9)	15 (2.8)	24 (18)	1 (1.7)	
Clinical N stage, *n* (%)					
cN0	710 (97)	522 (96.7)	130 (97.7)	58 (98.3)	0.669
cN1	22 (3)	18 (3.3)	3 (2.3)	1 (1.7)	
Biopsy grade group, *n* (%)					
1	343 (46,9)	262 (48.5)	54 (40.6)	27 (45.8)	0.030
2–3	315 (43)	229 (42.4)	66 (49.6)	20 (33.9)	
4–5	74 (10.1)	49 (9.1)	13 (9.8)	12 (20.3)	
Risk class, *n* (%)					
Low	250 (34.2)	193 (35.7)	37 (27.8)	20 (33.9)	0.079
Intermediate	367 (50.1)	271 (50.2)	72 (54.1)	24 (40.7)	
High	115 (15.7)	76 (14.1)	24 (18)	15 (13)	
**Pathological factors**					
Prostate weight, g, median (IQR)	50 (41–63)	52 (44–63.6)	51 (40.2–63.7)	50 (41.2–28.5)	0.226
Pathological grade group, *n* (%)					
1	126 (17.2)	107 (19.8)	15 (11.3)	4 (6.8)	<0.001
2–3	463 (63.3)	350 (64.8)	82 (61.7)	31 (52.5)	
4–5	143 (19.5)	83 (15.4)	36 (27.1)	24 (40.7)	
Pathological T stage, *n* (%)					
pT2	572 (78.1)	453 (83.9)	91 (68.4)	28 (47.5)	<0.001
pT3a	77 (10.5)	43 (8)	20 (15)	14 (23.7)	
pT3b	83 (11.4)	44 (8.1)	22 (16.5)	17 (28.8)	
Pathological N stage, *n* (%)					
pNx	390 (53.3)	302 (55.9)	57 (42.9)	31 (52.5)	0.003
pN0	293 (40)	212 (39.3)	61 (45.9)	20 (33.9)	
pN1	49 (6.7)	26 (4.8)	15 (11.3)	8 (16.3)	
**Perioperative factors**					
OT, min, median (IQR)	200 (160–240)	195 (160–230)	210 (165–245)	210 (155–250)	0.054
Blood loss, mL, median (IQR)	300 (200–500)	300 (200–500)	300 (200–450)	300 (200–500)	0.554
ePLND, *n* (%)					
No	390 (53.3)	302 (55.9)	57 (42.9)	31 (52.3)	0.026
Yes	342 (46.7)	238 (44.1)	76 (57.1)	28 (47.5)	
Nodes, *n*, median (IQR)	26 (21–33)	27 (21–34)	26 (22–32.7)	(19.2–28.5)	0.348
NSRP, *n* (%)					
No	87 (11.9)	63 (11.7)	21 (15.8)	3 (5.1)	0.163
Yes	600 (82)	440 (81.5)	106 (79.7)	54 (91.5)	
Unknown	45 (6.1)	37 (6.9)	6 (4.5)	2 (3.4)	
Surgeon volume, *n* (%)					
Low	248 (33.9)	168 (31.1)	57 (42.9)	23 (39)	0.026
High	484 (66.1)	372 (68.9)	76 (57.1)	36 (61)	
ASA score, *n* (%)					
1–2	675 (92.2)	495 (91.7)	124 (93.2)	56 (94.9)	0.601
3–4	57 (7.8)	45 (8.3)	9 (6.8)	3 (5.1)	
LOS, days, median (IQR)	4 (4–6)	4 (4–6)	4 (4–5)	5 (4–6)	0.069
Clavien–Dindo score, n (%)					
0	557 (76.1)	417 (77.2)	97 (72.9)	43 (72.9)	0.628
1–2	154 (21)	110 (20.4)	31 (23.3)	13 (22)	
>2	21 (2.9)	13 (2.4)	5 (3.8)	3 (5.1)	
Re-admission, *n* (%)					
No	711 (97.1)	522 (96.7)	132 (99.2)	57 (96.6)	0.271
Yes	21 (2.9)	18 (3.3)	1 (0.8)	2 (3.4)	

Overall (Table 1), 192 patients had PSMs (26.3%), which were classified as focal in 133 (18.2%) and non-focal in 59 (8.1%). Figure 2 shows the frequency of PSM sites stratified by linear extension amongst these patients. The predictors of PSMs and negative SMs in this cohort has been previously presented [30]. Considering clinical factors, significant associations of BMI, PSA level, BPC and biopsy grade group with focal and non-focal PSMs were detected. Amongst groups, the association was negative for BMI and positive for other factors. Amongst pathological factors, significant positive associations of extraprostatic disease, high-grade tumours and LNI with focal and non-focal PSMs were assessed. The presence of extraprostatic disease, high-grade tumours and LNI were positively associated with the risk of focal and non-focal PSMs (Table 1). Considering perioperative factors, performing ePLND was positively associated with the risk focal and non-focal PSMs, high surgeon volume was negatively associated with focal and non-focal PSMs.

We evaluated different multivariate models including clinical, pathological and perioperative factors (Table 2); moreover, combined models were also assessed (Table 2). Considering each model, the risk of focal PSM was predicted by BMI (odds ratio [OR] 0.919; *P* = 0.009) and BPC (OR 1.013; *P* = 0.005) in the clinical model, by extracapsular extension (OR 1.972; *P* = 0.027) in the pathological model; by ePLND (OR 1.675; *P* = 0.009) and high surgeon volume (OR 0.609; *P* = 0.013) in the perioperative model (Table 2). Moreover, in the clinical and perioperative models, focal PSMs were predicted by BMI (OR 0.915; *P* = 0.006), BPC (OR 1.012; *P* = 0.014) and high surgeon volume (OR 0.577; *P* = 0.007). In the overall model, BMI (OR 0.914; *P* = 0.006), BPC (OR 1.011; *P* = 0.015), pT3a (OR 2.064; *P* = 0.016), pT3b (OR 2.150; *P* = 0.010) and high surgeon volume (OR 0.574; *P* = 0.006) were associated with the risk of focal PSMs (Table 3).

**Table 2.** Multivariate models of factors associated with the risk of PSM in the 732 patients who underwent RARP.

	Focal vs negative SMs		Non focal vs negative SMs	
Variable	OR (95%CI)	*P*	OR (95%CI)	*P*
**Clinical model**				
BMI	0.919 (0.863–0.980)	0.009	0.981 (0.889–1.071)	0.369
PSA	1.012 (0.980–1.045)	0.464	1.018 (0.984–1.053)	0.304
BPC	1.013 (1.004–1.023)	0.005	1.018 (1.005–1.031)	0.005
Biopsy grade group 1	Ref.		Ref	
Biopsy grade group 2–3	1.216 (0.805–1.835)	0.353	0.753 (0.406–1.398)	0.369
Biopsy grade group 4–5	0.994 (0.488–2.024)	0.994	1.617 (0.726–3.603)	0.240
**Pathological model**				
Pathological grade group				
1	Ref.		Ref.	
2–3	1.527 (0.841–2.773)	0.164	1.864 (0.634–5.475)	0.258
4–5	1.952 (0.921–4.139)	0.081	3.128 (0.935–10.454)	0.064
Pathological T stage				
pT2	Ref.		Ref.	
pT3a	1.972 (1.081–3.599)	0.027	4.089 (1.924–8.690)	<0.001
pT3b	1.773 (0.932–3.375)	0.081	4.074 (1.845–8.998)	0.001
Pathological N stage				
pN0–x	Ref.		Ref.	
pN1	1.574 (0.745–3.325)	0.235	1.156 (0.448–2.981)	0.764
**Perioperative model**				
ePLND				
No	Ref.		Ref.	
Yes	1.675 (1.140–2.461)	0.009	1.138 (0.664–1.952)	0.638
Surgeon volume				
Low	Ref.		Ref.	
High	0.609 (0.412–0.900)	0.013	0.709 (0.407–1.134)	0.224

**Table 3.** Combined multivariate models of factors associated with the risk of PSM in the 732 patients who underwent RARP.

	Focal PSM vs negative SMs		Non-focal PSM vs negative SMs	
Variable	OR (95%CI)	*P*	OR (95%CI)	*P*
**Clinical/perioperative model**				
BMI	0.915 (0.859–0.975)	0.006		
BPC	1.012 (1.002–1.022)	0.014	1.021 (1.009–1.033)	<0.001
ePLND				
No	Ref.			
Yes	1.338 (0.878–2.039)	0.176		
Surgeon volume				
Low	Ref.			
High	0.577 (0.388–0.859)	0.007		
Final overall model*				
BMI	0.914 (0.857–0.974)	0.006		
BPC	1.011 (1.002–1.021)	0.015	1.013 (1.000–1.023)	0.044
Pathological T stage				
pT2	Ref.		Ref.	
pT3a	2.064 (1.145–3.722)	0.016	4.832 (2.348–9.943)	<0.001
pT3b	2.15 (1.196–3.864)	0.010	5.153 (2.541–10.450)	0.001
Surgeon volume				
Low	Ref			
High	0.574 (0.385–0.855)	0.006		

*adjusted ORs.

The risk of non-focal PSM was predicted only by BPC (OR 1.018; *P* = 0.005) in the clinical model; by extracapsular extension (OR 4.089; *P* < 0.001) and seminal vesicle invasion (OR 4.074; *P* = 0.001) in the pathological model; and by no factor in the perioperative model (Table 2). In the overall model, BPC (OR 1.013; *P* = 0.044), extracapsular extension (OR 4.832; *P* < 0.001) and seminal vesicle invasion (OR 5.153; *P* = 0.001) were associated with the risk of non-focal PSMs (Table 3).

Considering differences between PSM subgroups, BMI and high surgeon volume were the only predictors that were associated with the risk of focal PSM. Considering, similarities between PSM subgroups, BPC, pT3a and pT3b were factors that were associated with the risk of both focal and non-focal PSMs.

In Table 4, we stratified the SM status by tumour pathological stage and compared them between low- and high-volume surgeons. The high-volume surgeon had better oncological results when compared to low-volume surgeons (15.7% vs 23%). When stratified by tumour stage, the difference was substantial for pT2 (13.6% vs 20.5%) and pT3a stage (19.1% vs 36.7%).

**Table 4.** SM status stratified by tumour stage in low- and high-volume surgeons.

Variable, *n* (%)	Subpopulation (*)	SM status (**)		
		Negative	Focal PSM	Non-focal PSM
**Low-volume surgeons**				
Pathological tumour stage				
pT2	190 (76.6)	141 (74.2)	39 (20.5)	10 (5.3)
pT3a	30 (12.1)	12 (40)	11 (36.7)	7 (23.3)
pT3b	28 (11.3)	15 (53.6)	7 (25)	6 (21.4)
Total	248	168 (67.7)	57 (23)	23 (9.3)
**High-volume surgeon**				
Pathological tumour stage				
pT2	382 (78.9)	312 (81.7)	52 (13.6)	18 (4.7)
pT3a	47 (9.7)	31 (66)	9 (19.1)	7 (14.9)
pT3b	55 (11.4)	29 (52.7)	15 (27.3)	11 (20)
Total	484	372 (76.9)	76 (15.7)	36 (7.4)

Legend: percentages are relative to columns (*) or rows (**).

## Discussion

PSM rates after RARP in contemporary series range from 15% to 29.5% [31–37]; with the PSM rate in our present study (26.2%) consistent with these series. We showed that PSM can be evaluated according to the linear extent of cancer in a simple and effective way, with focal PSMs detected more frequently (18.2%) than non-focal patterns (8.1%). The extent of cancer involving the SM is a feature that should be considered when counselling patients after RP.

PSM after RP represents an unfavourable outcome, which increases the risk of biochemical recurrence (BCR). The length of the PSM is also an increasingly negative prognostic factor [3–10]. Although measurement of the whole linear extent of a PSM is suggested [7], it is not routinely applied in clinical practice because it is time consuming [3–10]. Patients with non-focal PSMs are expected to have higher rates of BCR than those with focal patterns, and the risk of biochemical persistence is higher for the former [38–40]. Patients with non-focal PSMs are at increased risk of a second treatment after RARP and this is a critical issue that should be extensively explained when counselling patients [38–40]. Different thresholds have been used in the literature to describe focal PSM. Particularly, Servoll et al. [38] have shown that a PSM length >3 mm was an independent predictor of cancer recurrence in 303 patients who underwent open RP. Sammon et al. [39] used 1 mm as a threshold in 794 patients undergoing perineal RP and they found that at a median follow-up of 54 months, the 5-year BCR-free probability was 90.8% in patients with negative SMs, 77.5% in patients with focal (<1 mm) PSMs, and 47.5% in patients with broad (>1 mm) PSMs. Lee et al. [40] demonstrated that focal (<3 mm) PSMs after RP does not significantly affect BCR-free survival in 1733 patients with prostate cancer. Because RARP was performed in our present cohort, we have chosen 1 mm as our threshold and evaluated the linear extent of PSM in two groups that were simple to compute and allowed a division of the population of patients into two subsets.

It is important to identify predictive factors of PSMs in clinical practice, in order to define the risk, to plan the surgery, and to anticipate the potential need for adjuvant treatments after surgery. Therefore, patents who are at risk of PSM need appropriate counselling before and after RP due to the risk of needing adjuvant or salvage treatments [3]. Detecting a PSM after RP is an unfavourable outcome, which is dependent on both surgery and tumour biology [3,4,6–10,13]. The former is related to technique and surgeon’s experience, whilst the latter is dependent on tumour stage, Gleason grade, and prostatic microenvironment. In this context, in our previous experience, we found that higher preoperative serum testosterone levels were predictive of PSMs after RP [41]. The risk of PSMs after RARP has been associated with various clinical and pathological factors [31–33]. Clinical predictors include: BMI, PSA level, prostate volume, BPC, biopsy grade group, and extraprostatic disease. Pathological factors are characterised by cancer extending beyond the prostate (pT3a and pT3b stage), PSA level, prostate volume, BPC, biopsy grade group, cT and pT stage, are factors that relate to tumour extension, which increases the risk of PSMs during prostate dissection. Further, in our previous experience, we found that higher preoperative serum testosterone levels were predictive of PSMs after RP [41]. The association of BMI with the overall risk of PSMs is a controversial topic, where published data have found both that there is no association and a positive association [33,42,43]. Patel et al. [33] suggest that a positive association between BMI and PSMs could be related to both reduced vision and angle movements during RARP in obese patients. A further step is to identify predictors of the linear extent of PSM and also factors predicting BCR [10,26]. This issue is lacking in the literature.

Our present study showed that other factors were able to differentiate the linear extent of PSM beyond BPC and pT stage (Table 2; overall multivariate model).

High BMI and high surgeon volume were both independent factors that were associated with a reduced risk of focal PSMs. The influence of BMI during RARP is unclear. We previously found that BMI is associated with major postoperative complications after RARP [44]. In the present study, we found that BMI was independently associated with a reduced risk of focal but not non-focal PSMs, and this represents a new finding. This association may be related to periprostatic fat tissue thickness, which is more substantial in obese patients and thus they are less likely to have focal PSM during RARP. Although this hypothesis needs to be verified, it is supported by a study showing a significant correlation between BMI and periprostatic fat thickness (*r* = 0.37), which was measured by CT [40]. In the present study, we compared RARP performed by five experienced surgeons. One surgeon had performed >500 RARPs, whilst the other four low-volume surgeons had performed between 50 and 60 procedures. In the literature there is no consensus about the number of the procedures a surgeon needs to complete in order to reduce the PSM rate. Atug et al. [45] demonstrated in the 2006 that experience gained over time led to a decrease in the incidence of PSM. In that study a reduction in PSM was found after ~30 procedures, but the PSMs were not stratified according to their extension. In our experience all surgeons overtook this number of procedures when the evaluation of patients started and we found only a correlation with focal PSMs (≤1 mm). In this context, the experience of the surgical team should be discussed with the patients during preoperative counselling.

A systematic review of the literature investigated the subject of surgeon volume and oncological outcomes [46]. The review found that overall oncological outcomes are improved by increasing surgeon volume. Hu et al. [47] reported that patients who underwent RARP by high-volume surgeons were less likely to undergo salvage therapy after RARP. Moreover, Steinsvik et al. [48] found that the overall risk of PSM after RARP was reduced in patients undergoing RP by high-volume surgeons. Our present study showed that high surgeon volume specifically and independently decreased the risk of focal PSM, which is a new finding. We stratified the SM status by pathological tumour stage and compared low- and high-volume surgeons (Table 4). For the issue of focal PSM between surgeons, we found that the high-volume surgeon had better oncological results than the low-volume surgeons (15.7% vs 23%). When stratifying by tumour stage, the difference was substantial for pT2 (13.6% vs 20.5%) and pT3a stage (19.1% vs 36.7%). Our present results show that surgeon volume is an independent factor that reduces the risk of focal PSM in high-volume centres; this is helpful during the learning curve because it alerts low-volume surgeons to focus more on the quality of the their surgery, as well as stimulating high-volume surgeons to improve the quality of their surgery to decrease the rate of focal PSM in pT2 and pT3a disease. Additionally, concerned patients, especially those with more aggressive disease may seek high-volume surgeons in high-volume centres in order to avoid the consequences of a suboptimal RARP. Also, our present results may further contribute to the notion that RARP should be limited to high-volume surgeons at high-volume centres. Referring physicians should consider this when counselling patients before RARP.

Our present study has some limitations. First, the retrospective nature of the present study is a limitation in itself. Second, prostate volumes and biopsies performed elsewhere were not re-evaluated; however, their features had good standard quality to support their analysis. Third, in some cases the pathologists did not report the location, but just evaluated the linear extension of the PSM. Fourth, we did not provide a threshold for defining high- and low-volume surgeons due to the presence of a large difference between the single high- and four low-volume surgeons and the homogenous experience of the low-volume surgeons. For these reasons, we compared the cohort of the patients who were operated on by the single high-volume surgeon with the cohort of the patients who were operated on by the other four low-volume surgeons. However, beyond these limits, our present study has much strength as it includes a large contemporary cohort of patients and our dedicated pathologists assessed all specimens.

## Conclusions

In high-volume centres features related to host, tumour and surgeon volume are important factors that are associated with the risk of focal and non-focal PSMs after RARP. Particularly, high-volume surgeons have lower focal PSMs than low-volume surgeons. These issues should be discussed when counselling patients.
